# Analysis of Latency Performance of Bluetooth Low Energy (BLE) Networks

**DOI:** 10.3390/s150100059

**Published:** 2014-12-23

**Authors:** Keuchul Cho, Woojin Park, Moonki Hong, Gisu Park, Wooseong Cho, Jihoon Seo, Kijun Han

**Affiliations:** 1 School of Computer Science and Engineering, Kyungpook National University, Daegu 702-701, Korea; E-Mails: k5435n@netopia.knu.ac.kr (K.C.); kspark@netopia.knu.ac.kr (G.P.); wscho@netopia.knu.ac.kr (W.C.); jhseo87@netopia.knu.ac.kr (J.S.); 2 Software R&D Center, Samsung Electronics Co., Ltd., Suwon 443-742, Korea; E-Mails: woojin1.park@samsung.com (W.P.); moonki1.hong@samsung.com (M.H.)

**Keywords:** Internet of Things, Bluetooth 4.0, Bluetooth low energy, discovery

## Abstract

Bluetooth Low Energy (BLE) is a short-range wireless communication technology aiming at low-cost and low-power communication. The performance evaluation of classical Bluetooth device discovery have been intensively studied using analytical modeling and simulative methods, but these techniques are not applicable to BLE, since BLE has a fundamental change in the design of the discovery mechanism, including the usage of three advertising channels. Recently, there several works have analyzed the topic of BLE device discovery, but these studies are still far from thorough. It is thus necessary to develop a new, accurate model for the BLE discovery process. In particular, the wide range settings of the parameters introduce lots of potential for BLE devices to customize their discovery performance. This motivates our study of modeling the BLE discovery process and performing intensive simulation. This paper is focused on building an analytical model to investigate the discovery probability, as well as the expected discovery latency, which are then validated via extensive experiments. Our analysis considers both continuous and discontinuous scanning modes. We analyze the sensitivity of these performance metrics to parameter settings to quantitatively examine to what extent parameters influence the performance metric of the discovery processes.

## 1. Introduction

Bluetooth Low Energy (BLE) is a new wireless communication technology for short-range communication with enhanced low-cost and low-power properties. A major and fundamental change has been made in the BLE radio architecture to enable short-range communication in BLE [1–4]. BLE has a very low power consumption rate with a similar communication range. Devices that use BLE for communication are normally powered by coin-cell batteries and it can be operated for months and even for years [1,2].

BLE technology provides support of novel data transfer functionality and hence it can be used in sensor technologies for transmitting bulk data [3]. The BLE technology offers a more advanced and robust connectivity by re-establishing the connections with the devices once they come back into the range of each other. This novel data transfer functionality make BLE more favorable for short-range communication in wireless sensor networks.

Recently, most technologies are using the ISM band for communication and hence it has become more congested and crowded, therefore, BLE uses a frequency-hopping mechanism in both its advertising and data channels to avoid congestion during communication. The classical Bluetooth has 79 channels, each of which has a width of 1 MHz, while the BLE is designed to operate on 2.4 GHz ISM band using 40 channels, each with a width of 2 MHz. Out of these 40 channels, three channels, that is channels number 37, 38, and 39, are used for broadcasting purposes, *i.e.*, device discovery, *etc.*, and the remaining 37 channels are responsible for data transmission. The device discovery process is more simplified by designing a concise state-machine which also helps in supporting the power saving functionality [3,5].

These properties of BLE make it more favorable for short-range communication technologies. The Bluetooth Special Interest Group (SIG) recommended a number of markets for BLE technology such as Body Area Networks (BAN) and Internet of Things (IoT), these includes automotive, consumer electronics, health issues and wellness, sports activities and fitness, and smart homes [6].

Since BLE is developed for these short-range wireless applications, a fast and convenient discovery process becomes as one of the important feature which can be addressed and attached to the existing BLE technologies. The BLE standard clearly elaborated and published the communication process but many areas still need to be studied and researched such as the device discovery latency and energy efficiency of the system [4]. The BLE technology recommended a wide range of parameter settings for the device discovery mechanism and its proper tuning to balance and optimize the performance for a wide range of applications in context of latency and energy consumption [3].

Keeping these challenges in mind, this paper focuses on the discovery process of BLE networks, and an analytical model is proposed to investigate the discovery probability, as well as expected discovery latency, which are then validated via extensive simulation experiments. In addition, we also analyze the sensitivity of those performance metrics to quantitatively evaluate to what extent parameter setting would influence the performance metrics.

The rest of this paper is organized as follows: related work covering the classical Bluetooth standards and BLE device discovery process is presented in Section 2. A comprehensive detailed overview of BLE and its discovery mechanism are provided in Section 3. An analytical model for the BLE process is presented in Section 4. Section 5 validates our model, and finally conclusions are given in Section 6.

## 2. Related Works

Recently, the device discovery performance of classical Bluetooth protocols has been intensively investigated through real time experiments, simulations, and formal modeling methods [3]. The related work section is further divided into two parts, the first part explaining the performance evaluation of classical Bluetooth networks and second part explains the device discovery process of BLE in detail.

A detailed analysis on device discovery performance for classical Bluetooth Version 1.1 and 1.2 has been presented in [7]. The probabilistic model checking technique and the PRISM tool were used to compute the performance bounds of device discovery in terms of the mean time and the mean power consumption [3]. Their study has proved that a low-level analysis can produce exact results like those derived from simulation techniques, but if the analysis is performed on a high number of nodes it can produce insignificant results and thus it can be applied in a congested environment.

A comprehensive experiment on real devices, exploring the parameter space to determine the relationship between parameter settings and mean discovery latency or power consumption values has been proposed in [8]. An algorithm is proposed to adaptively determine parameter settings, depending on a mobility context to reduce the mean power consumption for Bluetooth devices. The tradeoff between different parameters is not clearly explained. It looks like that by increasing the value of one parameter a significant change can be seen in another parameter. Thus the work proposed in that research cannot be applied to the next generation networks like IoT and BAN. Similarly, a simulative study on device discovery in multi-hop Bluetooth networks, *i.e.*, Bluetooth Scatternet, by means of classical Bluetooth inquiry procedure has been addressed in [9]. Different types of experiments were performed to show that even though it required a long time for each node to become aware of all its neighbors, the Bluetooth topologies can be obtained in about 6 s after the connection setup through those discovered devices [3]. This amount of time is very high for applications where a fast topology construction is important.

In [10], the authors implemented an end-to-end Bluetooth-based mobile service framework. The framework relied on machine-readable visual tags for out-of-band device and service selection rather than using the standard Bluetooth device discovery model to detect nearby mobile services. Their work demonstrated that a tag-based connection establishment technique could offer significant improvements over the standard Bluetooth device discovery model. Although there have been intensive studies presented for classical Bluetooth device discovery, but unfortunately, these studies cannot be applied to BLE, since the Bluetooth standard made a fundamental change in the device discovery mechanism of BLE. Very little research work related to the performance evaluation of the BLE discovery process has been published. Some literature covering the BLE discovery mechanism is reviewed in the following section.

An analytical model for device discovery in BLE networks by developing a new BLE extension accounting for all the protocols based on original Bluetooth and its validation through simulation results in NS-2 was proposed in [3]. They compared the analytical results with those obtained through simulation [3]. Since intermittent connections are frequently encountered in practical BLE scenarios, the modeling results can provide a beneficial guidance to customize the advertising or scanning behavior towards the required performance [5]. The model can be used to determine some important performance metrics, such as mean latency or mean energy consumption during the course of discovering neighbors, but many parameters and metrics bust bed define and tested for the implementation of said proposed scheme in real world scenarios.

In [11], the authors introduced an analytical model, based on classical ALOHA analysis, to investigate two metrics, such as discovery latency and connection setup latency, in WBAN applications. The probability of successful device discovery was also computed. They studied the performance of BLE device discovery, particularly with multiple devices. The average latency of device discovery is given by:

$${\text{D}}_{CS}=\left(\frac{1}{{\text{P}}_{CS}}-1\right)\text{R}+{\text{T}}_{S}+{\text{T}}_{\text{IFS}}+{\text{T}}_{CR}$$

where T_S_, T_CR_, and T_IFS_ denote the sending time of ADV_IND and CONN_REQ packets, and inter frame space, respectively, derived from the length over bit rate of R bps (44 octets over 1 Mbps), and P_CS_ means the successful probability of the connection setup. The modeling results as well as the methodology may provide a potential guide to better enhance the performance of the BLE advertising process. In addition, they proposed an algorithm using the so-called connection report for BLE scanner to perceive the network contention degree and adaptively adjust its scanning parameters, so as to achieve shorter latency. The complexity of the proposed scheme is very high and it requires high energy for the scanning process. Energy consumption is an important constraint in the case of BLE implementation for short-range communications and hence it should be considered carefully.

In [3], the authors focused on the modeling and performance of the device discovery process in BLE networks. A general model for device discovery in multi-channel scenarios was proposed primarily, and then the model was tailored and simplified for the BLE network with three broadcasting channels. The average discovery latency was derived through theoretical analysis. They revealed that improper parameter settings can significantly deteriorate the device discovery latency and increase meaningless energy consumption. They consequently proposed a solution to adaptively reduce the discovery latency when encountering an exceptionally long delay to be discovered by any scanner. Based on that, they devised three different strategies which significantly enhanced the latency performance regarding to the parameter settings. Through extensive simulation, they validated the accuracy of the model, showing the effectiveness of their strategies in overcoming the traps of the standard towards fast and efficient device discovery in BLE networks.

The previous studies on BLE discovery are still far from thorough. Since intermittent device discovery is commonplace in BLE networks, it is important to know to what extent parameter setting would influence the discovery process [3,4,7]. In fact, wide-range settings of the parameters provide new features for BLE devices to customize their performance in specific applications [2,5,11]. In other words, an advertiser should be capable of selecting appropriate parameters that meet the requirements for practical BLE networks. It is thus necessary to develop a new, accurate discovery model for existing BLE architectures. This motivates our study of modeling the discovery process of BLE and performing an intensive simulative evaluation.

## 3. Background of BLE Discovery

### 3.1. Operation of BLE

**Figure 1.**
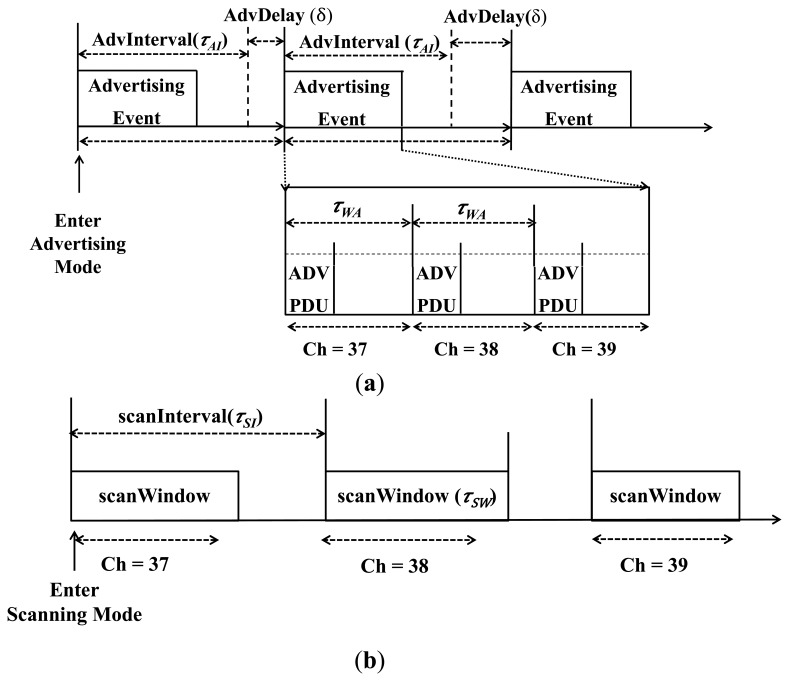
Advertising and scanning process for device discovery [5]. (**a**) Advertising process; (**b**) Scanning process.

A BLE device may operate in three different modes depending on required functionality: advertising, scanning and initiating, as shown in Figure 1. A device in advertising mode, named advertiser, periodically transmits advertising information in three advertising channels (index = 37, 38, 39) [3]. As shown in Figure 1a, an advertiser keeps sending ADV_IND Packet Data Units (PDUs) in sequence over each of the three advertising channels in advertising event, which is composed by a fixed AdvInterval (hereafter denoted by τ_AI_) and a pseudo-random AdvDelay (hereafter denoted by δ) generated by the Link Layer [2,3]. The random variety AdvDelay to the advertising interval is used to separate the advertisement interval when two or more advertisers are getting close [3]. Since BLE advertisers set the time randomly between consecutive advertising PDUs, advertisings on the three channels become completely asynchronous, then the successful advertising probability will reach an optimum value [3,11]. If all advertisers are set with the same advertising interval between consecutive advertising PDUs, then collisions on the first channel will pass to the second and the third channels [3,11].

Generally, there are two kinds of advertising events for BLE: undirected and directed. The undirected advertising event contains ADV_IND, which is used for detecting unknown devices yet allows different responses [11]. Different from undirected events, the directed advertising event is used for establishing connections with already known devices. It contains just one PDU type ADV_DIRECT_IND, and has no defined random delay between advertising events [11]. According to the standard, the AdvInterval should be an integer multiple of 0.625 ms in the range of 20 ms to 10.24 s, the AdvDelay should be within the range of 0 ms to 10 ms. According to the specification, an advertisement period for each channel (denoted by τ_WA_) shall be less than or equal to 10 ms [11]. After each sending of the advertising packets, the advertiser will be listening on the same channel for a while to check if there is a response coming from any scanner [4].

On the other hand, a BLE device in scanning mode, named scanner, periodically scans the advertising channels and listens to advertising information of advertisers [5]. On receiving an advertising channel packet, the scanner will send back a response. As shown in Figure 1b, by each ScanInterval (denoted by τ_SI_) the scanner scans on a different advertising channel for the duration of ScanWindow (denoted by τ_SW_) [5]. According to the standard, the ScanInterval and ScanWindow should be less than or equal to 10.24 s. Table 1 shows the list of major timing parameters specified in BLE standard.

The scanner shares a similar process except that they can only respond to specific types of advertising packets. For example, the scanner responds to ADV_SCAN_IND PDU by transmitting a SCAN_REQ to request additional information of the advertiser. If the advertiser receives a SCAN_REQ that contains its device address from a scanner allowed by the advertising filter policy, it shall reply with SCAN_RSP PDU on the same advertising channel index [2,5]. Hereinafter, however, we generically refer to both ADV_SCAN_IND and ADV_DIRECT_IND just as “ADV_IND” since the distinction between them is irrelevant to our analysis. In addition, since multiple scanners may respond to an advertiser simultaneously, back-off procedures are used by each scanner to minimize collision [5].

### 3.2. Types of BLE PDU

Some details about packets related to discovery are presented in terms of the format and the length, which are important factors for discovery analysis. A BLE link layer packet has four components: preamble, access address, PDU and Cyclic Redundancy Check (CRC). The PDU has different types, and is further composed of a header and a payload. The packet length is decided by the length of the payload, ranging from 0 octets to 37 octets [3].

**Figure 2.**
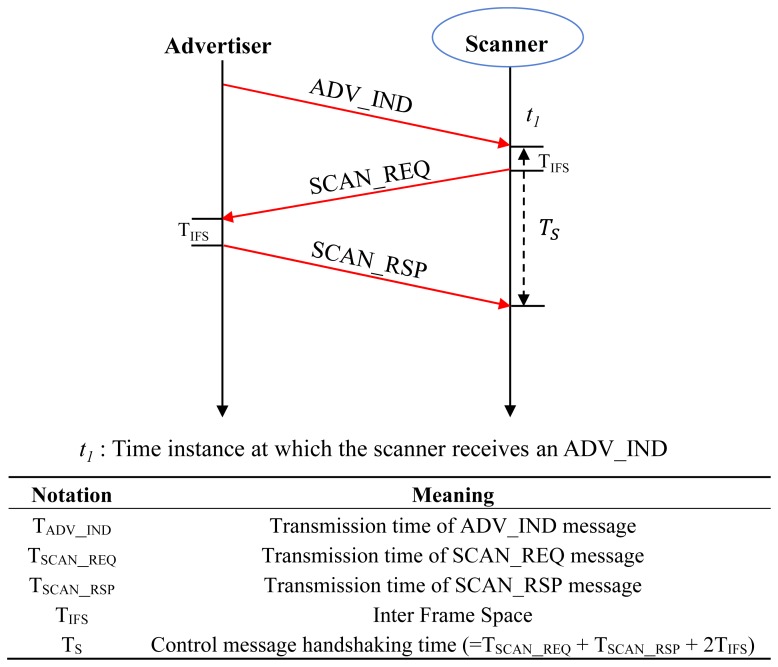
Control messages for discovery process and their transmission times.

As previously described, the advertiser sends an ADV_IND over each advertising channel and is listening on the same channel to respond to SCAN_REQ from any scanner. The scanning procedure is defined as an operation where the scanner replies a SCAN_REQ PDU upon receiving an ADV_IND from the advertiser on the same advertising channel. The time needed for handshaking control messages in the scanning procedure is denoted by T_S_ (=T_SCAN_REQ_ + T_SCAN_RSP_ + 2T_IFS_) as shown in Figure 2. Figure 2 shows the list of PDU transmission times and handshaking times for exchanging control messages between the advertiser and the scanner for device discovery, which are derived from the length of each PDU over bit rate (1 Mbps).

## 4. Analytical Model

We investigate the performance of the BLE device discovery from the perspective of theoretical model in full accordance with the BLE specification to investigate performance metrics. We first present an analytical model for the probability of device discovery, which will be used as a basis to derive an analytical model for mean discovery latency. The sensitivity index is evaluated to what extent parameter settings influence those performance metrics. The proposed analytical analysis is derived on investigating the cases where a particular pair of the advertiser and scanner (called A_1_ and S_1_) successfully discover each other and establish a connection between them. Different from other wireless networks, BLE exploits three advertising channels and employs tiny-sized frames, and advertisers do not examine the channel state before transmission, that is, the medium is accessed in a completely unsynchronized manner [4].

**Figure 3.**
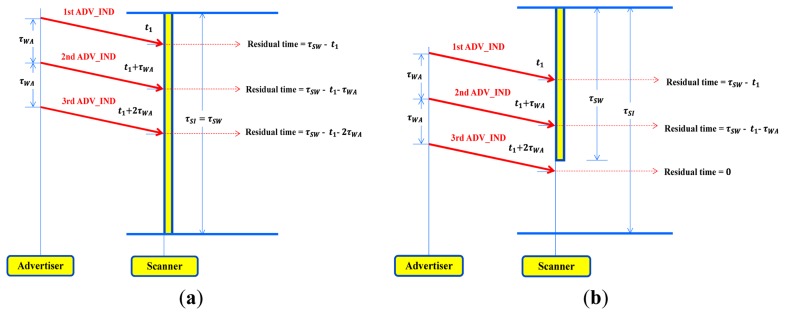
Continuous scanning (τ*_SI_* = τ*_SW_*) and discontinuous scanning (τ*_SI_* > τ*_SW_*) (**a**) A continuous scanning without sleeping (τ*_SI_* = τ*_SW_*); (**b**) Discontinuous scanning (τ*_SI_* > τ*_SW_*).

According to BLE standard, an advertiser sends an ADV_IND at the beginning of each advertising period (denoted by τ*_WA_*) per advertising channel. The advertiser changes its advertising channel in a circular way (37→38→39→37…) with a period of τ*_WA_*, and the scanner also changes its scanning channel in the same way every ScanInterval (with a period of τ*_SI_*). We consider both continuous and discontinuous scanning modes to build analytical models. In the continuous scanning mode, BLE device scans each advertising channel without sleeping (therefore, τ*_SW_* = τ*_SI_*) as shown in Figure 3. On the contrary, the discontinuous scanning refers to a mode in which BLE device alternatively repeats scanning and sleeping every ScanInterval. So, τ*_SW_* should be shorter than τ*_SI_* in the discontinuous scanning mode as illustrated in Figure 3. We introduce a duty cycle to express how long a BLE device spends time on scanning process during a given ScanInterval. The duty cycle is defined as the proportion of time during which a BLE device is waking up for scanning, and is denoted by ρ = τ*_SW_*/τ*_SI_*. There is no doubt that 0 ≤ ρ ≤ 1. In particular, ρ becomes equal to one in the continuous scanning mode.

It should be noted that τ*_WA_* is usually much longer than the handshaking time needed to exchange control packets for discovery. For example, the advertiser spends about 0.6 ms to exchange SCAN_REQ and SCAN_RSP between any scanner on each advertising channel for device discovery, so we can assume that the advertiser A_1_ has enough time to exchange control packets after sending an ADV_IND to successfully discover S_1_ and establish a connection.

### 4.1. Probability of Successful Discovery

We define six events to clearly express under what conditions advertiser A_1_ can successfully discover S_1_, as listed in Table 2. Using these events, we can derive situations where A_1_ can successfully discover S_1_.

#### 4.1.1. 1:1 Network

In order to clearly elaborate the proposed analytical model, considering a network, in which one device acts as an advertiser and other works as a scanner. For successful discovery, the advertiser should rendezvous with the scanner at one of three advertising channels (37, 38, and 39). This case can be expressed by E1 ∩ E4 using events in Table 2.

**Figure 4.**
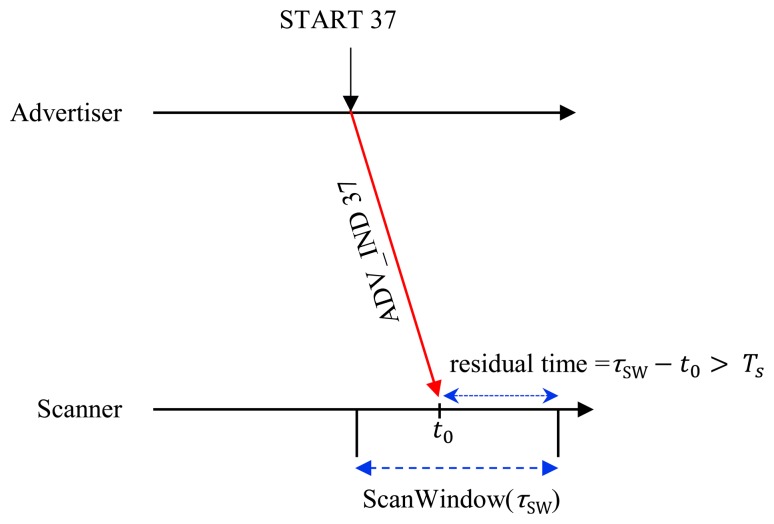
Case (E1 ∩ E4) for successful discovery in 1:1 network.

At the same time, the scanner should have a sufficient residual time to interact with the advertiser by handshaking of control messages after receiving an ADV_IND, so the advertiser can successfully discover the scanner if the following case is satisfied as shown in Figure 4.

The probability that the scanner rendezvous with the advertiser on receipt of the first ADV_IND is given by C31(13)2⋅C31 is the probability of selecting a specific channel out of three channels (37, 38 and 39). Assuming that the scanner receives the first ADV_IND at an arbitrary time instance of *t*_0_, the residual time until completion of ScanWindow is given by (τ*_SW_* − *t*_0_). For successful discovery, the scanner should reply to ADV_IND with a SCAN_REQ message and should receive a SCAN_RSP from the advertiser within the residual time. In other words, the scanner should have a sufficient residual time greater than T_S_ (=T_SCAN_REQ_ + T_SCAN_RSP_ + 2T_IFS_) until completion of ScanWindow for exchanging control messages with the advertiser. The probability that there is sufficient residual time is given by $\frac{\tau_{SW}-{\text{T}}_{S}}{\tau_{SI}}$ under assumption of continuous scanning scenario. So, we have the probability of successful discovery on the first advertising channel *α*1,by:

(1) α1=PROB(E1∩E4)=C31(13)2{ρ−(TSτSI)}

Since the advertiser sends another ADV_IND on the next advertising channel after a duration of τ*_WA_*, the scanner receives the second ADV_IND at*t*_0_ + τ*_WA_*, so, the residual time for the second ADV_IND until completion of ScanWindow is (*a* τ*_SI_* + τ_SW_ − *t*_0_ − τ*_WA_*) where $\text{a}=\left\lfloor \frac{\tau_{WA}}{\tau_{SI}}\right\rfloor$ (⌊*x*⌋ means the largest integer not greater than *x*) as shown in Figure 4. The probability that the scanner has enough time for handshaking control messages for the second ADV_IND until completion of ScanWindow is given by $\left(\frac{a\tau_{SI}+\tau_{SW}-\tau_{WA}-{\text{T}}_{S}}{\tau_{SI}}\right)$. Since the probability that the scanner rendezvous with the advertiser on receipt of the second ADV_IND is given by C31(13)2, we have the probability of discovery successful on the second advertising channel by:

(2) $$\alpha_{2}=\left(\frac{1}{3}\right)\left(a+\rho -\frac{\tau_{WA}}{\tau_{SI}}-\frac{T_{S}}{\tau_{SI}}\right)$$

Similarly, the arrival time of the third ADV_IND at the scanner is *t*_0_ + 2τ*_WA_*, and thus the residual time for the third ADV_IND until completion of the ScanWindow is given by (*b*τ*_SI_* + τ*_SW_* − *t*_0_ − 2τ*_WA_*) where $\text{b}=\left\lfloor \frac{2\tau_{WA}}{\tau_{SI}}\right\rfloor$. Thus, we have the probability of discovery successful on the third advertising channel by:

(3) $$\alpha_{3}=\left(\frac{1}{3}\right)\left(b+\rho -\frac{2\tau_{WA}}{\tau_{SI}}-\frac{T_{S}}{\tau_{SI}}\right)$$

We can rewrite Equations (1)–(3) into a more compact form as follows:

(4) $$\alpha_{k}=\left(\frac{1}{3}\right)\left(\left\lfloor \left(k-1\right)\frac{\tau_{WA}}{\tau_{SI}}\right\rfloor +\rho -\left(k-1\right)\frac{\tau_{WA}}{\tau_{SI}}-\frac{T_{S}}{\tau_{SI}}\right)\;,\left(k=1,2,3\right)$$

If τ*_SI_* = τ*_WA_*, we can see that α_1_ = α_2_ = α_3_ from Equation (4).

#### 4.1.2. 1:2 Networks

**Figure 5.**
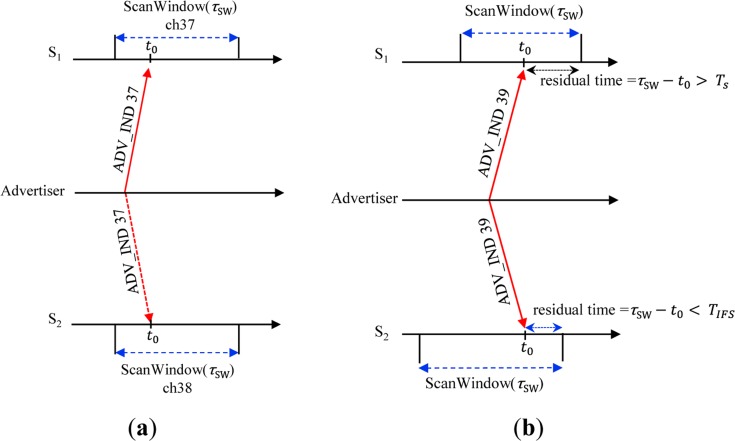
Cases for successful discovery in 1:2 networks (**a**) Case 2 (E1 ∩ E2 ∩ E4)); (**b**) Case 3 ( $\text{E}1\cap \bar{\text{E}2}\cap \text{E}4\cap \text{E}5$).

Assume that there are three BLE devices in the network, and one device acts as an advertiser and the other two (called S_1_ and S_2_) work as scanners. In this case, the advertiser can successfully discover the scanner S_1_ if one of the following cases is satisfied as shown in Figure 5:

We can easily get the probability of case 2 by:

(5) PROB(E1∩E2∩E4)=C31(13)2(23){ρ−(TSτSW)}

The advertiser and two scanners can rendezvous at one of three advertising channels with a probability of C31(13)3. The probability that only S_1_ has a sufficient residual time for handshaking control messages is given by $\frac{\tau_{SW}-\tau_{S}}{\tau_{SI}}$. And, the probability that S_2_ cannot send any message since its residual time is shorter than T*_S_* is given by $\left(\frac{{\text{T}}_{\text{IFS}}}{\tau_{SI}}\right)$. So, we get the probability of case 3 by:

(6) PROB(E1∩E2¯∩E4∩E5)=C31(13)3(τSW−TSτSI)(TIFSτSI)

where $\bar{\text{E}2}$ means the complementary event of E2.

Now, we have:

(7) $$\alpha_{k}={\left(\frac{1}{3}\right)}^{2}\left(\left\lfloor \left(k-1\right)\frac{\tau_{WA}}{\tau_{SI}}\right\rfloor +\rho -\left(k-1\right)\frac{\tau_{WA}}{\tau_{SI}}-\frac{T_{S}}{\tau_{SI}}\right)\left(3-\rho +\frac{T_{\text{IFS}}}{\tau_{SI}}\right)\left(k=1,2,3\right)$$

where $a=\left\lfloor \frac{\tau_{WA}}{\tau_{SI}}\right\rfloor$ and $b=\left\lfloor \frac{2\tau_{WA}}{\tau_{SI}}\right\rfloor$ as previously defined.

#### 4.1.3. M:N Network

**Figure 6.**
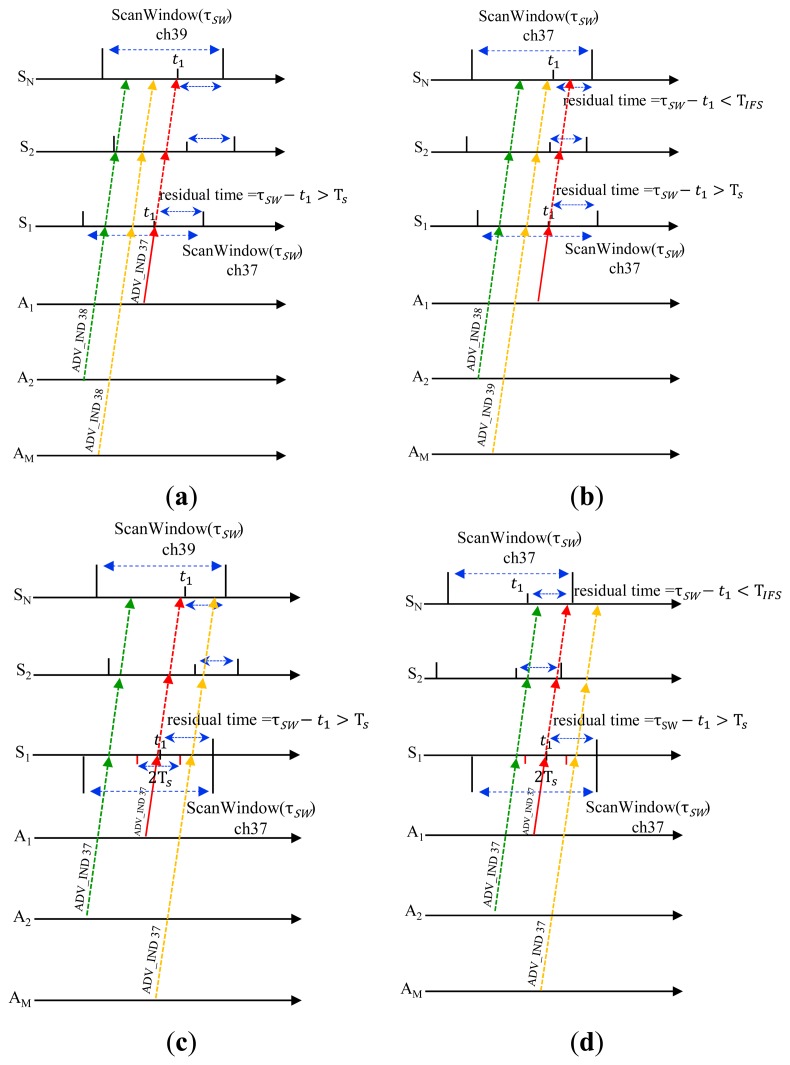
Cases for successful discovery in M:N network. (**a**) Case 4 (E1 ∩ E2 ∩ E3 ∩ E4); (**b**) Case 5 ( $\text{E}1\cap \bar{\text{E}2}\cap \text{E}3\cap \text{E}4\cap \text{E}5$); (**c**) Case 6 ( $\text{E}1\cap \text{E}2\cap \bar{\text{E}3}\cap \text{E}4\cap \text{E}6$); (**d**) Case 7 ( $\text{E}1\cap \bar{\text{E}2}\cap \bar{\text{E}3}\cap \text{E}4\cap \text{E}5\cap \text{E}6$).

Now, we inspect a more general case for a network with (N+M) BLE devices, where M devices (called A_1_, A_2_, …, A_M_) act as advertisers and the other N devices (called S_1_, S_2_, …, S_N_) work as scanners. In this case, the advertiser A_1_ can successfully discover S_1_ if one of four cases shown in Figure 6 is satisfied.

In the same way as previously, we get:

(8) PROB(E1∩E2∩E3∩E4)=C31(13)2(23)M+N−2(τSW−TSτSI)=(13)(23)M+N−2(ρ−TSτSI)PROB(E1∩E2¯∩E3∩E4∩E5)=C31(13)2(23)M−1(τSW−TSτSI){∑k=1N−1CN−1k(13)k(τSI−τSW−TIFSτSI)k(23)N−1−k}=(13)N(23)M−1(ρ−TSτSI)[(3−ρ+TIFSτSI)N−1−2N−1]

(9) PROB(E1∩E2∩E3¯∩E4∩E6)=C31(13)2(23)N−1(τSW−TSτSI){∑k=1M−1CM−1k(13)k(τSI−2TSτSI)k(23)M−1−k}=(13)M(23)N−1(ρ−TSτSI)[(3−2TSτSI)M−1−2M−1]

(10) PROB(E1∩E2¯∩E3¯∩E4∩E5∩E6)=C31(13)2(τSW−TSτSI){∑k=1M−1CM−1k(13)k(τSI−2TSτSI)k(23)M−1−k}{∑k=1N−1CN−1k(13)k(τSI−τSW+TIFSτSI)k(23)N−1−k}

(11) $$={\left(\frac{1}{3}\right)}^{M+N-1}\left(\rho -\frac{{\text{T}}_{S}}{\tau_{SI}}\right)\left[{\left(3-\frac{2{\text{T}}_{S}}{\tau_{SI}}\right)}^{M-1}-2^{M-1}\right]\;\left[{\left(3-\rho +\frac{{\text{T}}_{\text{IFS}}}{\tau_{SI}}\right)}^{N-1}-2^{N-1}\right]$$

Summing Equations (8)–(11), we have the probability of successful discovery on the first advertising channel by:

(12) $$\alpha_{1}={\left(\frac{1}{3}\right)}^{M+N-1}\left(\rho -\frac{{\text{T}}_{S}}{\tau_{SI}}\right){\left(\text{3}-\frac{{\text{2T}}_{S}}{\tau_{SI}}\right)}^{M-1}{\left(\text{3}-\rho +\frac{{\text{T}}_{\text{IFS}}}{\tau_{SI}}\right)}^{N-1}$$

The above equation becomes identical to Equation (4), by substitute *M* = 1 and *N* = 1, respectively, in Equation (12). Similarly, we can get the probability of successful discovery on the second and third advertising channel, respectively, by

(13) $$\alpha_{k}={\left(\frac{1}{3}\right)}^{M+N-1}\left(\left\lfloor \left(k-1\right)\frac{\tau_{SW}}{\tau_{SI}}\right\rfloor +\rho -\left(k-1\right)\frac{\tau_{WA}}{\tau_{SI}}-\frac{T_{S}}{\tau_{SI}}\right){\left(3-\frac{2T_{\text{S}}}{\tau_{SI}}\right)}^{M-1}{\left(3-\rho +\frac{T_{\text{IFS}}}{\tau_{SI}}\right)}^{N-1}\left(\text{k}=1,2,3\right)$$

### 4.2. Expected Discovery Latency

**Figure 7.**
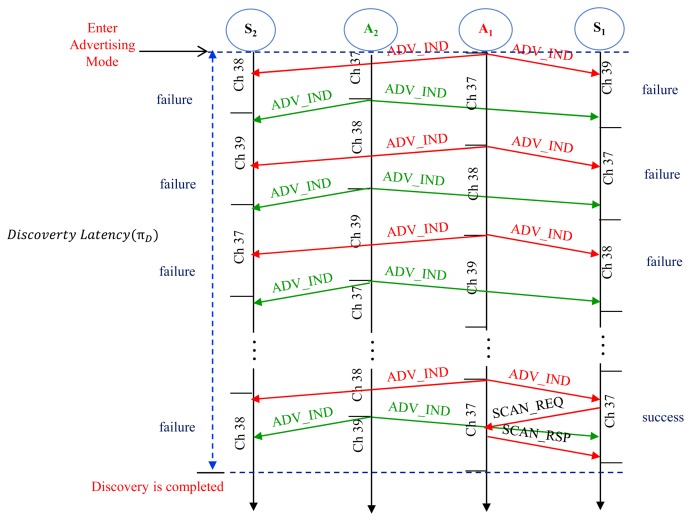
Discovery latency.

The discovery latency is defined as the interval for the advertiser from entering into the first advertising event by sending an ADV_IND until it successfully receives a SCAN_REQ from the scanner as illustrated in Figure 7. The time to successful discovery depends on the number of failures experienced in attempts during the discovery process as shown in Table 3, where α_1_, α_2_, α_3_ means the probability of successful discovery on the first, the second, and the third advertising channel, respectively, as previously discussed.

From Table 3, we can get the expected discovery latency, denoted by π*_D_*, by:

(14) $$\begin{array}{c} \pi_{D}=\sum_{i=1}^{\infty }{\left(1-\alpha_{1}\right)}^{i-1}{\left(1-\alpha_{2}\right)}^{i-1}{\left(1-\alpha_{3}\right)}^{i-1}\alpha_{1}\left\{\left(i-1\right)\left(\tau_{AI}+\frac{\delta_{\max}}{2}\right)+\tau_{WA}\right\}\\ +\sum_{i=1}^{\infty }{\left(1-\alpha_{1}\right)}^{i}{\left(1-\alpha_{2}\right)}^{i-1}{\left(1-\alpha_{3}\right)}^{i-1}\alpha_{2}\left\{\left(i-1\right)\left(\tau_{AI}+\frac{\delta_{\max}}{2}\right)+2\tau_{WA}\right\}\\ +\overset{\infty }{\underset{i=1}{\text{∑}}}{\left(1-\alpha_{1}\right)}^{i}{\left(1-\alpha_{2}\right)}^{i}{\left(1-\alpha_{3}\right)}^{i-1}\alpha_{3}\left\{\left(i-1\right)\left(\tau_{AI}+\frac{\delta_{\max}}{2}\right)+3\tau_{WA}\right\} \end{array}$$

Using algebra $\sum_{i=0}^{\infty }ix^{i}=x\frac{d}{dx}\left(\sum_{i=0}^{\infty }x^{i}\right)=\frac{x}{{\left(1-x\right)}^{2}}$, we get:

(15) $$\begin{array}{c} \pi_{D}=\left[\frac{\left(1-\alpha_{1}\right)\left(1-\alpha_{2}\right)\left(1-\alpha_{3}\right)}{1-\left(1-\alpha_{1}\right)\left(1-\alpha_{2}\right)\left(1-\alpha_{3}\right)}\right]\left(\tau_{AI}+\frac{\delta_{\max}}{2}\right)\\ +\left[\frac{1+\left(1-\alpha_{1}\right)+\left(1-\alpha_{1}\right)\left(1-\alpha_{2}\right)-3\left(1-\alpha_{1}\right)+\left(1-\alpha_{2}\right)\left(1-\alpha_{3}\right)}{1-\left(1-\alpha_{1}\right)\left(1-\alpha_{2}\right)\left(1-\alpha_{3}\right)}\right]\tau_{WA} \end{array}$$

The expected discovery latency of M:N networks can be determined using Equation (13) respectively, by substituting the corresponding probability of successful discovery in Equation (15).

### 4.3. Sensitivity Analysis

In order to investigate to what extent parameter setting influences the performance metrics, The sensitivity index (*S_Q_*_,_*_x_*) is defined as follows:

(16) $$S_{Q,x}=\frac{\left(\frac{\Delta Q}{Q}\right)}{\gamma }$$

where *x* and *Q* mean a parameter and a performance metric, respectively, and Δ*Q* means the change in the performance metric Q when the value of the parameter *x* is changed by a factor of γ. In other words, Δ*Q* means how much the performance metric Q will be changed when the value of the parameter *x* is changed from a value h to h(1 + *γ*), so Δ*Q*/*Q* indicates the ratio of change in the performance metric Q as the value of the parameter *x* is changed from h to h(1 + *γ*). Thus, the sensitivity index *S_Q_*_,_*_x_* indicates the extent to how much a performance metric Q is affected as the value of a parameter x is changed. In our analysis, the value of γ is given by −0.5 < γ < 0.5. A larger value of *S_Q_*_,_*_x_* indicates a stronger sensitivity. For example, if *S_Q_*_,_*_x_* = 2.0, it means that the performance metric Q is highly affected by x as much as double of γ. If *S_Q_*_,_*_x_* = 1.0, it means that Q is affected by *x* in the same ratio as γ. If *S_Q_*_,_*_x_* = 0.5, it means that Q is influenced by *x* as much as half of γ. In that case, Q is relatively insensitive to parameter x. On the other hand, if *S_Q_*_,_*_x_* = −0.5, it means that Q is inversely affected by *x* as much as the half of γ. If *S_Q_*_,_*_x_* = −1.0, it means that Q is inversely affected by *x* in the identical ratio as γ. In this subsection, we present sensitivity of performance metrics in 1:N network as an example.

By substitution of τ_SW_ with τ_SW_(1 + γ) and subtraction in Equation (13), we can get Δα_1_ by:

(17) $$\begin{array}{c} \Delta \alpha_{1}={\left(\frac{1}{3}\right)}^{N}\left\{\frac{\tau_{SW}\left(1+\gamma \right)}{\tau_{SI}}-\frac{T_{S}}{\tau_{SI}}\right\}{\left\{3-\frac{\tau_{SW}\left(1+\gamma \right)}{\tau_{SI}}-\frac{T_{\text{IFS}}}{\tau_{SI}}\right\}}^{N-1}\\ -{\left(\frac{1}{3}\right)}^{N}\left(\frac{\tau_{SW}}{\tau_{SI}}-\frac{T_{S}}{\tau_{SI}}\right)\left(3-\frac{\tau_{SW}}{\tau_{SI}}+\frac{T_{\text{IFS}}}{\tau_{SI}}\right) \end{array}$$

Thus, the sensitivity of discovery probability (S_α1,_*_SW_*) becomes:

(18) $$S_{\alpha_{1},SW}=\frac{\left(\frac{\Delta \alpha_{1}}{\alpha_{1}}\right)}{\gamma }$$

We can also find the sensitivity of the expected discovery latency to τ_AI_ in M:N network using Equations (13) and (15):

(19) $$S_{D,AI}=\frac{\left(\frac{\Delta \pi_{D}}{\pi_{D}}\right)}{\gamma }=\frac{\left(1-\alpha_{1}\right)\left(1-\alpha_{2}\right)\left(1-\alpha_{3}\right)}{1-\left(1-\alpha_{1}\right)\left(1-\alpha_{2}\right)\left(1-\alpha_{3}\right)}$$

Similarly, the sensitivity of the expected discovery latency to other parameters is obtained such as τ_SW_ and τ_WA_ in M:N network using Equations (13) and (15), but we do not present the final expressions of S*_D_*_,_*_SW_* and S*_D_*_,_*_WA_* since they have very complicated forms since α_1_, α_2_, α_3_ are functions of τ_SW_ and τ_WA_ as seen in Equation (13).

## 5. Simulation Validation

In order to validate the analytical models, we have developed a BLE simulation program which fully complies with the BLE specification. The simulative settings are in accordance with the standard definition as previously described, and we compare the analytical results with those obtained via simulations. We simulate over 10 times for each scenario to get the average results, where parameter settings are selected with values listed in Table 4.

**Figure 8.**
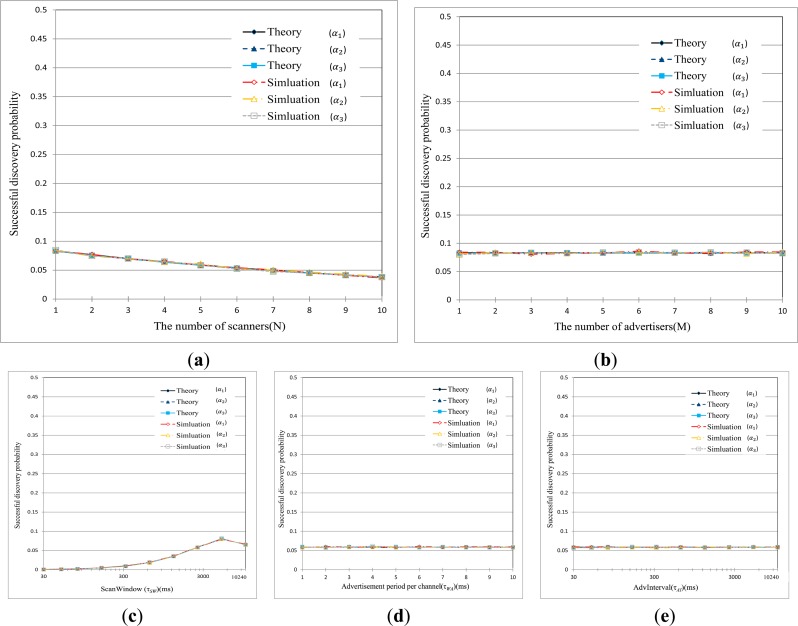
Probability of successful discovery on each advertising channel with various parameter settings. (**a**) The probability of successful discovery as the number of scanners increase (τ*_SI_* = 10,240, τ*_SW_* = 2560, τ*_WA_* = 10, τ*_AI_* = 1280, M = 5); (**b**) the probability of successful discovery as the number of advertisers increase (τ*_SI_* = 10240, τ*_SW_* = 2560, τ*_WA_* = 10, τ*_AI_* = 1280, N = 5); (**c**) the probability of the successful discovery as the ScanWindow (τ*_SW_*) is varied is (τ*_SI_* = 10,240, τ*_WA_* = 10, τ*_AI_* = 1280, M = 5, N = 5); (**d**) the probability of the successful discovery as τ*_WA_* is varied (τ*_SI_* = 10,240, τ*_SW_* = 2560, τ*_AI_* = 1280, M = 5, N = 5); (**e**) the probability of the successful discovery as AdvInterval (τ*_AI_*) is varied (τ*_SI_* = 10,240, τ*_SW_* = 2560, τ*_WA_* = 10, M = 5, N = 5).

Figures 8 show the results of performance evaluation relating to the discovery probability, respectively, in terms of different sets of parameters such as τ_AI_, τ_WA_, and τ_SW_ in BLE network. The graphs are obtained by varying one parameter while setting the other two parameters to their default values listed in Table 4. From these figures, it is first found the theoretical curves practically coincide with the simulation results over the entire range of parameters. We can see that the discovery probability is very low, although there are not so many devices in the network. For example, the scanner experiences the success probability of about 0.3 in discovering process in even 1:1 network, which is totally different from the behavior of other wireless networks. This is because BLE devices can be synchronized with one of three advertising channels with a probability of 1/3 to discover each other. Further, as the number of devices increases in the network, the discovery process fails more frequently due to collision of the abundant control packets, such as SCAN_REQ, SCAN_RSP, during discovery process.

It can be seen from following graphs, the discovery probability is somewhat comparatively affected by τ_WA_ and τ_SW_. The scanning duration is only dependent on τ_SW_ and the number of scans per advertisement is determined by the ratio of τ_SW_ to τ_WA_. As τ_SW_ increases, the probability that the scanner successfully discovers the advertiser increases very gradually when τ_SW_ < 100 ms, and remains almost constant when τ_SW_ exceeds 100 ms. This is because the scanner can stay a long time on each channel for scanning in spite of decreasing number of scans as τ_SW_ is increased. So, we can say that the scanner loses many chances of device discovery if τ_SW_ is shorter than the expectation needed by the advertiser. In particular, if τ_WA_ = τ_SW_, α_2_ and α_3_ becomes identical to α_1_ which implies that discovery on the first channel will pass to the second and the third channel.

On the contrary, the discovery probability is not affected by τ*_AI_* since this parameter influences neither scanning duration nor the number of scans. Instead, τ*_AI_* is only used to determine when the advertiser initiates the next advertisement process. Figures 8 indicates that inappropriately setting of parameters significantly deteriorates the successful device discovery, respectively.

**Figure 9.**
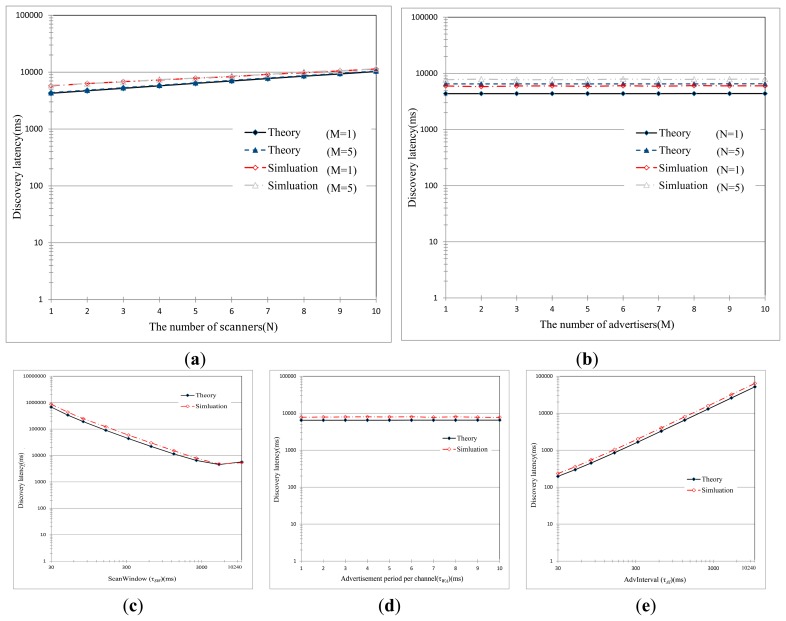
Mean discovery latency with various parameter settings. (**a**) The mean discovery latency as the number of scanners is increased (τ*_SI_* = 10,240, τ*_SW_* = 2560, τ*_WA_* = 10, τ*_AI_* = 1,280); (**b**) the mean discovery latency as the number of advertisers increase (τ*_SI_* = 10,240, τ*_SW_* = 2560, τ*_WA_* = 10, τ*_AI_* = 1280); (**c**) the mean discovery latency as ScanWindow (τ_SW_) is varied (τ*_SI_* = 10,240, τ*_WA_* = 10, τ*_AI_* = 1280, M = 5, N = 5); (**d**) the mean discovery latency as τ*_WA_* is varied (τ*_SI_* = 10,240, τ*_SW_* = 2560, τ*_AI_* = 1280, M = 5, N = 5); (**e**) the mean discovery latency as AdvInterval (τ*_AI_*) is varied (τ*_SI_* = 10,240, τ*_SW_* = 2560, τ*_WA_* = 10, M = 5, N = 5).

Figure 9 shows the mean discovery latency in terms of different sets of parameters, such as τ*_AI_*, τ*_WA_*, and τ*_SW_*. We can find that the theoretical results match with the simulative ones over the entire range of parameters. The low success probability, as seen in Figures 8, causes a significant and exponential rise in the mean discovery latency as the number of BLE devices increased. As explained above, this is because of collisions of the abundant control packets. From the figure, an interesting thing can be discovered. The mean discovery latency decreases very gradually with τ*_SW_* when τ*_SW_* < 100 ms, and remains almost constant when τ*_SW_* > 100 ms. And, the mean discovery latency is not affected much by τ*_WA_* (a negligible change though). As previously described, τ*_SW_* and τ*_WA_* mainly affect the rendezvous chance of the advertiser and the scanner on one of three advertising channels, but they do not have a significant impact on the discovery latency. On the contrary, the mean discovery latency linearly increases with τ*_AI_*. Since advertisers initiate the advertisement intervals according to the value of τ*_AI_*, the discovery latency is strongly dependent on τ*_AI_*. Figure 9 also indicates that the improper setting of parameters significantly deteriorates the discovery latency.

**Figure 10.**
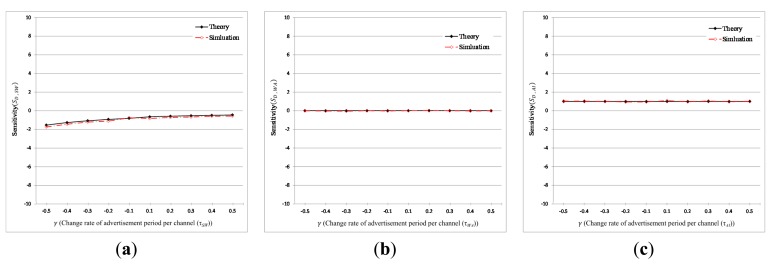
Sensitivity of mean discovery latency with various parameter setting. (**a**) The sensitivity of mean discovery latency to τ*_SW_* (τ*_SI_* = 10,240, τ*_WA_* = 10, τ*_AI_* = 1280, M = 5, N = 5); (**b**) the sensitivity of mean discovery latency to τ*_WA_* (τ*_SI_* = 10,240, τ*_SW_* = 2560, τ*_AI_* = 1280, M = 5, N = 5); (**c**) the sensitivity of mean discovery latency to τ*_AI_* (τ*_SI_* = 10,240, τ*_SW_* = 2560, τ*_WA_* = 10, M = 5, N = 5).

As for the sensitivity shown in Figure 10, it is shown that the theoretical results are practically the same with simulation results. The sensitivity of the mean discovery latency to τ_SW_ or τ_WA_ roughly remains around 0, which implies that change in τ_SW_ or τ_SW_ does not have a significant impact on the discovery latency. On the contrary, the sensitivity of discovery latency to τ_AI_ is almost 1 as *γ* is varied over a range from −0.5 to 0.5. This means that the discovery latency is identically proportional to τ_AI_, which is in accordance with the results shown in Figures 9 and 10.

## 6. Conclusions

There is a significant increase in the applications of BLE in different areas, which is capable of making BLE one of the leading technologies for short-range communication in the next generation of networks. The Bluetooth standard defined the BLE communication model in a clear and detailed way, but still there are many other parts which can be addressed. Therefore, we create an accurate analytical model for these parts such as the discovery latency, as well as the discovery probability in BLE networks. These are then validated via extensive simulation experiments. We also analyze the sensitivity of those performance metrics to quantitatively evaluate to what extent parameter setting would influence the performance metrics. It is shown the theoretical results match the simulated ones. With increasing number of BLE devices, delays of device discovery show an exponential growth despite the usage of three advertising channels and tiny-sized frames. This implies that there exist severe contentions among multiple BLE devices. We find that the inappropriate parameter settings considerably impair the efficiency of BLE devices, and the wide range of BLE parameters provides high flexibility for BLE devices to be customized for different applications. As far as we know, this work is one of the first in-depth and accurate models for BLE discovery, including sensitivity analysis.

## Figures and Tables

**Table 1.** List of major timing parameters.

**Notation**	**Meaning**	**Recommended Specification**
τ_WA_	Advertising period per channel (Max allowable waiting time for SCAN_REQ after sending ADV_IND on each channel)	≤10 ms
τ_AI_	Advertisement Interval for three advertising channels	Integer multiple of 0.625 ms in [20∼10,240] ms
δ	AdvDelay (Uniform random delay chosen from [0, δ_max_] to determine Advertisement Interval)	[0, δ_max_]
δ_max_	Upper bound to choose a random delay δ	≤10 ms
τ_SI_	Scan Interval	Integer multiple of 0.625 ms in [2.5∼10,240] ms
τ_SW_	Scan Window	Integer multiple of 0.625 ms in [2.5∼10,240] ms τ_SW_ ≤ τ_SI_

**Table 2.** Events used for analysis of the device discovery process.

**Event**	**Meaning**
E1	S_1_ is synchronous with A_1_
E2	All of S_2_, S_3_,… and S_N_ are not synchronous with A_1_
E3	All of A_2_, A_3_ …, and A_M_ are not synchronous with S_1_
E4	S_1_ has enough time to reply to ADV_IND until ScanWindow is finished
E5	All of S_2_, S_3_,… and S_N_ are sleeping or do not have enough time to reply to ADV_IND until ScanWindow is finished
E6	S_1_ does not receive ADV_IND from A_2_, A_3_ …, and A_M_ in an interval [t_1_ − T_s_, t_1_ + T_s_]

**Table 3.** Elapsed time to successful discovery and the corresponding probability.

**Advertising Interval**	**Channel ID**	**Elapsed Time to Successful Discovery**	**Probability of Successful Discovery**
1	1st	τ*_WA_*	α_1_
	2nd	2τ*_WA_*	(1 − α_1_)α_2_
	3rd	3τ*_WA_*	(1 − α_1_) (1 − α_2_)α_3_

2	1st	$\left(\tau_{AI}+\frac{\delta_{\max}}{2}\right)+\tau_{WA}$	(1 − α_1_) (1 − α_2_) (1 − α_3_)α_1_
	2nd	$\left(\tau_{AI}+\frac{\delta_{\max}}{2}\right)+2\tau_{WA}$	(1 − α_1_) (1 − α_2_) (1 − α_3_)α_2_
	3rd	$\left(\tau_{AI}+\frac{\delta_{\max}}{2}\right)+3\tau_{WA}$	(1 − α_1_)^2^ (1 − α_2_)^2^ (1 − α_3_)α_3_

…	…	…	…

I	1st	$\left(i-1\right)\left(\tau_{AI}+\frac{\delta_{\max}}{2}\right)+\tau_{WA}$	(1 − α_1_)*^i^* ^−1^ (1 − α_2_)*^i^* ^−1^ (1 − α_3_)*^i^* ^−1^α_1_
	2nd	$\left(i-1\right)\left(\tau_{AI}+\frac{\delta_{\max}}{2}\right)+2\tau_{WA}$	(1 − α_1_)*^i^* (1 − α_2_)*^i^* ^−1^ (1 − α_3_)*^i^* ^−1^α_2_
	3rd	$\left(i-1\right)\left(\tau_{AI}+\frac{\delta_{\max}}{2}\right)+3\tau_{WA}$	(1 − α_1_)*^i^* (1 − α_2_)*^i^* (1 − α_3_)*^i^* ^−1^α_3_

**Table 4.** Simulation parameters and their values.

**Parameters**	**Value**
Number of advertises (M)	1∼10
Number of scanners (N)	1∼10
τ_WA_	1∼10 (ms)
τ_AI_	30∼10,240 (ms)
δ_max_	10 (ms)
τ_SI_	30∼10,240 (ms)
τ_SW_	30∼10,240(ms)
T_ADV___IND_	0.128 (ms)
T_SCAN___REQ_	0.176 (ms)
T_SCAN___RSP_	0.128 (ms)
T_IFS_	0.150
T_S_	0.604
